# Variable importance analysis with interpretable machine learning for fair risk prediction

**DOI:** 10.1371/journal.pdig.0000542

**Published:** 2024-07-12

**Authors:** Yilin Ning, Siqi Li, Yih Yng Ng, Michael Yih Chong Chia, Han Nee Gan, Ling Tiah, Desmond Renhao Mao, Wei Ming Ng, Benjamin Sieu-Hon Leong, Nausheen Doctor, Marcus Eng Hock Ong, Nan Liu

**Affiliations:** 1 Centre for Quantitative Medicine, Duke-NUS Medical School, Singapore, Singapore; 2 Digital and Smart Health Office, Ng Teng Fong Centre for Healthcare Innovation, Singapore, Singapore; 3 Department of Preventive and Population Medicine, Tan Tock Seng Hospital, Singapore, Singapore; 4 Emergency Department, Tan Tock Seng Hospital, Singapore, Singapore; 5 Accident & Emergency, Changi General Hospital, Singapore, Singapore; 6 Department of Acute and Emergency Care, Khoo Teck Puat Hospital, Singapore, Singapore; 7 Emergency Medicine Department, Ng Teng Fong General Hospital, Singapore, Singapore; 8 Emergency Medicine Department, National University Hospital, Singapore, Singapore; 9 Department of Emergency Medicine, Sengkang General Hospital, Singapore, Singapore; 10 Programme in Health Services and Systems Research, Duke-NUS Medical School, Singapore, Singapore; 11 Department of Emergency Medicine, Singapore General Hospital, Singapore, Singapore; 12 Health Services Research Centre, Singapore Health Services, Singapore, Singapore; 13 Institute of Data Science, National University of Singapore, Singapore, Singapore; National Tsing-Hua University: National Tsing Hua University, TAIWAN

## Abstract

Machine learning (ML) methods are increasingly used to assess variable importance, but such black box models lack stability when limited in sample sizes, and do not formally indicate non-important factors. The Shapley variable importance cloud (ShapleyVIC) addresses these limitations by assessing variable importance from an ensemble of regression models, which enhances robustness while maintaining interpretability, and estimates uncertainty of overall importance to formally test its significance. In a clinical study, ShapleyVIC reasonably identified important variables when the random forest and XGBoost failed to, and generally reproduced the findings from smaller subsamples (n = 2500 and 500) when statistical power of the logistic regression became attenuated. Moreover, ShapleyVIC reasonably estimated non-significant importance of race to justify its exclusion from the final prediction model, as opposed to the race-dependent model from the conventional stepwise model building. Hence, ShapleyVIC is robust and interpretable for variable importance assessment, with potential contribution to fairer clinical risk prediction.

## Author summary

Logistic regression analyses are often used to quantify variable importance to clinical outcomes, which provide data-driven evidence for subsequent clinical interventions. However, such analyses are often affected by sampling variability, especially when sample sizes are small. Machine learning (ML) methods have been explored for robust alternatives for variable importance assessments, but they are often not interpretable, and lack stability with small sample sizes. These limitations could be addressed by the Shapley variable importance cloud (ShapleyVIC), which enhances regression-based variable importance analysis using ML approaches, hence improving robustness while maintaining interpretability. Another desirable property of ShapleyVIC is that it estimates the uncertainty of overall variable importance to exclude variables with non-significant importance to the prediction. In a clinical study, ShapleyVIC reasonably identified important variables when the random forest and XGBoost had questionable findings. For smaller subsamples (n = 2500 and 500), the logistic regression identified fewer important variables, but ShapleyVIC generally reproduced the findings from the full cohort. Moreover, ShapleyVIC reasonably identified the low importance of race and excluded it from the final prediction model, but the conventional model building approach failed to. Hence, ShapleyVIC is robust and interpretable for variable importance assessment, with potential contribution to fairer clinical risk prediction.

## Introduction

Understanding the impact of relevant factors on an outcome of interest is important in healthcare research, which generates evidence to inform intervention design and resource allocation. For example, the continuing efforts in understanding the impact of patient characteristics, emergency medical service (EMS) system and community interventions on outcomes (e.g., survival and neurological outcomes) after out-of-hospital cardiac arrest (OHCA) have contributed to improvements over the past decades via more responsive EMS systems, public cardiopulmonary resuscitation (CPR) training programs and beyond [1–3]. Existing evidence on variable importance to clinical outcomes is predominantly gathered using epidemiological and biostatistical approaches, e.g., from regression analyses of cohort data. Depending on budgets, study period and clinical topics of interest, sample sizes available may range from hundreds of thousands to just a few hundreds, and the associated sampling variability may partially contribute to inconsistencies in the resulting findings on variable importance, e.g., whether there are significant gender differences in OHCA outcomes to warrant further interventions [2–5].

With recent developments in machine learning (ML) methods and their successful applications in healthcare research [6], there is an increasing interest to update evidence from traditional regression analyses by using these advanced methods. In particular, ensemble methods such as the random forest (RF) [7] and XGBoost [8] have been well received in clinical applications for flexible non-linear structure and robust performance. Moreover, unlike other ML methods (e.g., neural network) that need additional analyses to quantify variable importance, RF and XGBoost provide built-in measures for convenient relative importance assessments. As an example, a recent study used the RF to confirm the current understanding of variable importance to OHCA survival and revisit the controversy around the importance of sex in the Swedish population [9].

Despite the good properties of aforementioned ML approaches, the use of such black-box methods to help understand the relationship between variables and the target outcome has been questioned and debated [10–12], and the sacrifice in interpretability may not bring clinically meaningful improvements [13]. Moreover, these ML approaches can only quantify the relative importance of variables, but such measures do not have any physical meaning or accompanying uncertainty intervals to conclude on the significance of importance, or to account for uncertainties in variable rankings due to sampling variability. In addition, such mathematically complex ML models often require larger sample sizes (usually thousands or more) for reliable model development compared to simpler regression models. This is especially limiting for clinical studies with small sample sizes, where conventional regression analyses will have limited statistical power to shortlist important factors, whereas ML-based inference may not be reliable. Hence, there remain unmet needs for robust and interpretable variable important assessments for clinical applications, especially when working with limited sample sizes. In this study, we demonstrate that this can be addressed by the Shapley variable importance cloud (ShapleyVIC) [14] method that enhances the familiar regression-based inference with the powerful ensemble approach in ML.

In view of the potentially inadequate (sometimes even biased) inference based on single models optimized on the data [14,15], ShapleyVIC evaluates variable importance with respect to an ensemble of regression models that performs nearly as well as the model with optimal performance, hence improving robustness while ensuring straightforward interpretation. Information is pooled across the ensemble to report an uncertainty interval for the overall importance of each variable, which helps to assess the statistical significance and to account for sampling variability when comparing variable importance. Previous studies have demonstrated the preferable properties of ShapleyVIC for identifying variables with non-significant importance for accurate predictions, but only with large datasets [14,16], and its value for small sample sizes have not been closely investigated. In this study, we highlight that the ensemble approach of ShapleyVIC can partially account for the loss in information with reduced sample sizes, making it useful for variable importance assessment for varying sample sizes. In a nationwide cohort study of OHCA survival in Singapore, we empirically demonstrate the less biased variable importance assessed using ShapleyVIC compared to regression or ML methods, and assess the stability of findings in simulated smaller subsamples. To further translate this robust assessment to fairer OHCA survival prediction, we illustrate a ShapleyVIC-assisted model building approach, and compare it with the widely used stepwise model building approach.

## Results

### Study cohort

In this study we analyzed variable importance to survival to discharge (or 30 days if not yet discharged) after OHCA among adult patients who had non-trauma etiology, were resuscitated and attained return of spontaneous circulation (ROSC), using information extracted from a nationwide data in Singapore. The final cohort analyzed in this study included 7490 OHCA patients with complete information on the outcome and 20 variables of interest, of which 1154 (15.4%) patients survived 30 days or to hospital discharge. Patient characteristics are described in Table 1.

**Table 1:** Patient characteristics of the full cohort.

	All (n = 7490)	Dead (n = 6336, 84.6%)	Alive (n = 1154, 15.4%)	P-value*
**Patient demographics**				
Age (median [Q1, Q3])	68 [57, 78]	69 [59, 79]	59 [50, 69]	<0.001
Gender (%)				<0.001
Female	2579 (34.4)	2337 (36.9)	242 (21.0)	
Male	4911 (65.6)	3999 (63.1)	912 (79.0)	
Race (%)				0.001
Chinese	5163 (68.9)	4373 (69.0)	790 (68.5)	
Indian	804 (10.7)	668 (10.5)	136 (11.8)	
Malay	1196 (16.0)	1040 (16.4)	156 (13.5)	
Others	327 (4.4)	255 (4.0)	72 (6.2)	
**OHCA incidence (%)**				
Cardiac etiology	4991 (66.6)	4098 (64.7)	893 (77.4)	<0.001
Witnessed arrest	5226 (69.8)	4276 (67.5)	950 (82.3)	<0.001
Shockable first rhythm	1980 (26.4)	1193 (18.8)	787 (68.2)	<0.001
Time of day				0.005
Day (06:00–18:59)	4796 (64.0)	4026 (63.5)	770 (66.7)	
Evening (19:00–22:59)	1336 (17.8)	1122 (17.7)	214 (18.5)	
Night (23:00–05:59)	1358 (18.1)	1188 (18.8)	170 (14.7)	
Arrest location				<0.001
Public	1729 (23.1)	1222 (19.3)	507 (43.9)	
Home	5097 (68.1)	4601 (72.6)	496 (43.0)	
Healthcare facility	664 (8.9)	513 (8.1)	151 (13.1)	
**Prehospital interventions (%)**				
Bystander CPR	3777 (50.4)	3063 (48.3)	714 (61.9)	<0.001
Bystander AED	568 (7.6)	351 (5.5)	217 (18.8)	<0.001
Mechanical CPR	4676 (62.4)	4211 (66.5)	465 (40.3)	<0.001
Prehospital defibrillation	2450 (32.7)	1658 (26.2)	792 (68.6)	<0.001
Prehospital advanced airway	6494 (86.7)	5800 (91.5)	694 (60.1)	<0.001
Prehospital medication	4291 (57.3)	3869 (61.1)	422 (36.6)	<0.001
Prehospital epinephrine	4146 (55.4)	3746 (59.1)	400 (34.7)	<0.001
**EMS system durations, minutes (median [Q1, Q3])**				
Response time	11.0 [9.0, 13.6]	11.1 [9.0, 13.7]	10.5 [8.5, 13.0]	<0.001
Call to ambulance dispatch	1.7 [1.1, 2.5]	1.7 [1.0, 2.5]	1.8 [1.2, 2.5]	<0.001
Ambulance dispatch to arrival at scene	6.6 [4.9, 8.7]	6.6 [5.0, 8.7]	6.3 [4.7, 8.1]	<0.001
Ambulance at scene	19.6 [15.4, 23.9]	19.8 [15.6, 24.0]	18.4 [14.0, 23.1]	<0.001
Ambulance leaving scene to arrival at ED	7.7 [5.0, 11.0]	7.7 [5.0, 11.0]	7.4 [4.7, 10.9]	0.094

*: p-values for comparison between patients with or without mortality, from the Chi-square test for categorical variables and Mann-Whitney test for continuous variables.

Q1, Q3: first- and third-quartile.

In the main analysis, we used the full cohort to compare ShapleyVIC with the logistic regression, RF and XGBoost for assessing variable importance, and demonstrated the benefit of ShapleyVIC-based variable importance for developing sparse and fair prediction model. The full cohort of 7490 patients was randomly divided into a training set of 60% observations (n = 4494) to evaluate variable importance and develop prediction models, and a test set of 40% observations (n = 2996) to evaluate predictive performance. To assess the stability of variable importance assessments with reduced sample sizes, we conducted simulated experiments with randomly generated samples of n = 2500 (approximately the sample size if restricted to two years in our cohort, e.g., to study specific interventions) and n = 500 (a feasible sample size for clinical studies under cost constraints). In the following subsections, we first summarize variable importance results from the main analysis and simulated experiments, and next describe the performance of prediction models from the main analysis.

### Variable importance assessment

**Fig 1:**
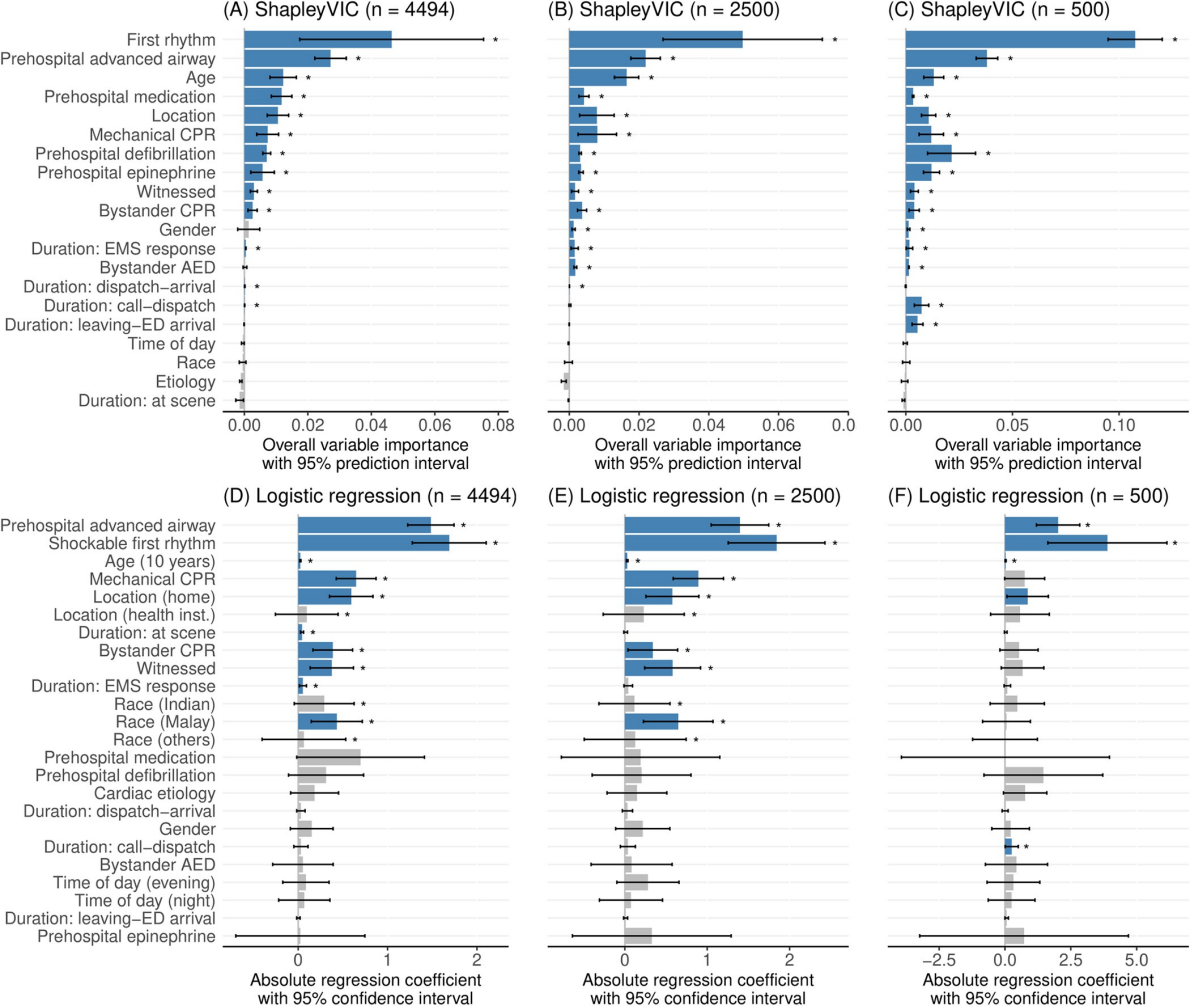
Variable importance analysis from ShapleyVIC (arranged by overall importance) and logistic regression analyses (arranged in ascending order of p-value), from the main analysis (n = 4494) and simulated experiments (n = 2500 and n = 500). Blue color and “*” indicate significant variable importance, i.e., 95% prediction interval above zero for ShapleyVIC and p-value<0.05 for logistic regression.

Fig 1A visualizes the overall importance and the uncertainty (assessed using the 95% prediction interval [PI]) of the 20 variables for accurate prediction of the outcome, assessed using ShapleyVIC from the training set of n = 4494 subjects. Thirteen of the 20 variables contributed significantly to the prediction, indicated by the 95% PI entirely above zero. First rhythm had higher overall importance than all other variables, followed by prehospital advanced airway, whereas the differences in overall importance were less evident among the next 8 variables (i.e., age to bystander CPR) in view of the largely overlapping 95% PIs. Although gender had the 11^th^ highest estimated overall importance among the 20 variables, it was statistically non-significant due to a wide 95% PI containing zero. Three duration variables (i.e., EMS response, ambulance dispatch to arrival, and call to ambulance dispatch) had low yet significant overall importance, and other variables (including race) had low and non-significant importance.

ShapleyVIC analysis of the simulated sample with n = 2500 subjects (see Fig 1B) were generally consistent with the findings from the main analysis, except for some changes in relative importance among the top 10 variables and some changes in significance for the other 10 variables with low importance (specifically gender, bystander automated external defibrillator [AED], and duration of ambulance leaving scene to emergency department [ED] arrival). When n = 500 (see Fig 1C), in addition to finding significance for gender and bystander AED, there were notable changes in the overall importance of prehospital medication, duration of call to ambulance dispatch and duration of ambulance leaving scene to ED arrival, but findings for other variables were generally consistent with those from the main analysis.

**Fig 2:**
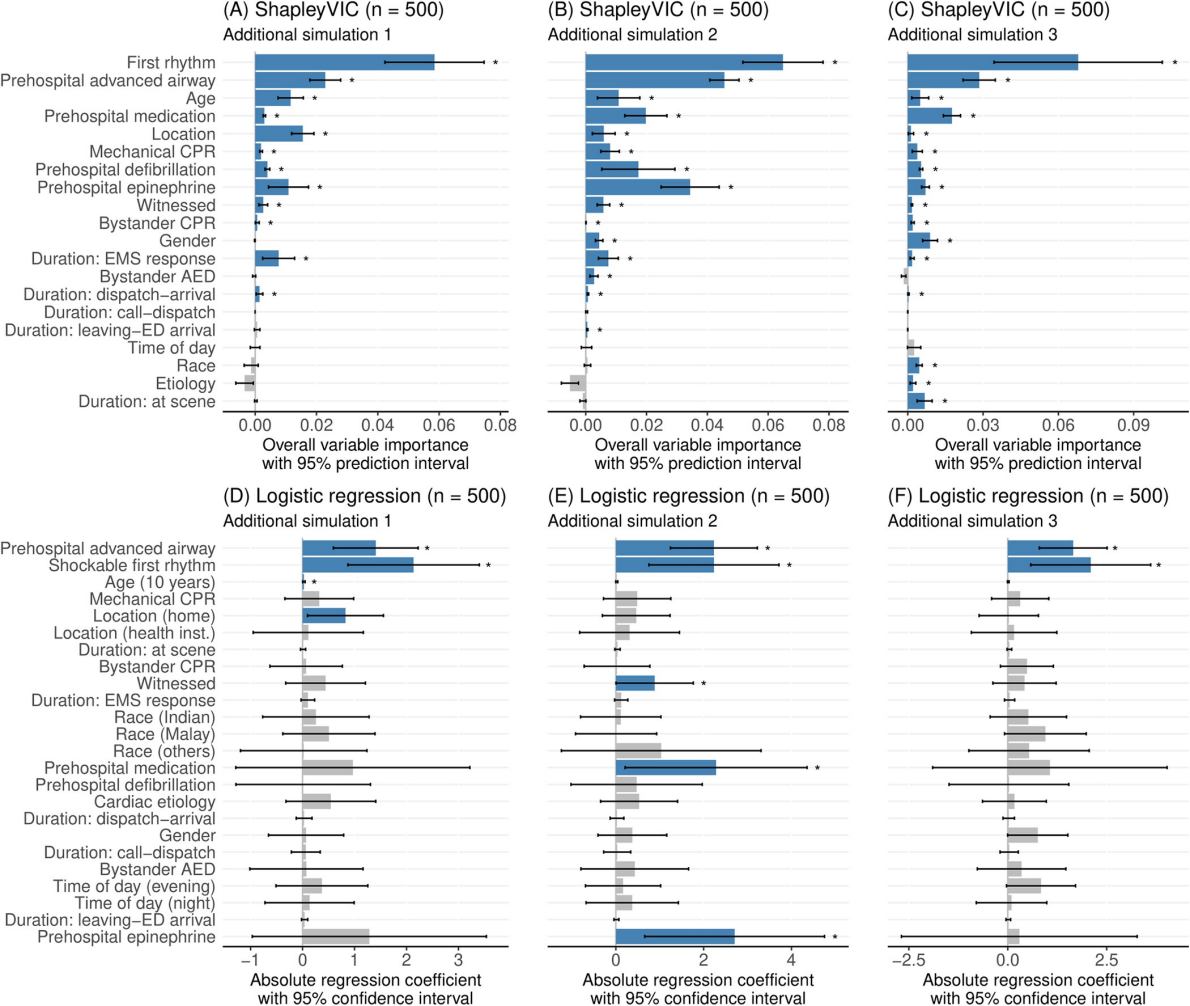
Variable importance analysis from ShapleyVIC and logistic regression analyses in 3 additional simulation experiments with n = 500, arranged by the same variable ordering as in Fig 1. Blue color and “*” indicate significant variable importance, i.e., 95% prediction interval above zero for ShapleyVIC and p-value<0.05 for logistic regression.

The conventional multivariable logistic regression analysis of the training data found 10 variables were significantly associated with the outcome (based on p-value<0.05; see Fig 1D), of which 8 variables had significant importance in the ShapleyVIC analysis. Specially, unlike in the ShapleyVIC analysis, the logistic regression analysis reported significant importance for race and duration of ambulance at scene, and non-significance for all prehospital interventions except for advanced airway. When sample size reduced to n = 2500 (see Fig 1E), the 7^th^- and 9^th^-ranking variables in the main analysis became non-significant, unlike ShapleyVIC findings where changes in significance were only observed for lower-ranking variables. When n = 500 (see Fig 1F), only four variables had p-value<0.05. As shown in three additional simulation experiments with n = 500, ShapleyVIC was generally able to shortlist the important variables identified from the main analysis (see Fig 2A–2C), whereas the logistic regression could only consistently shortlist prehospital advanced airway and shockable first rhythm (see Fig 2D–2F).

**Fig 3:**
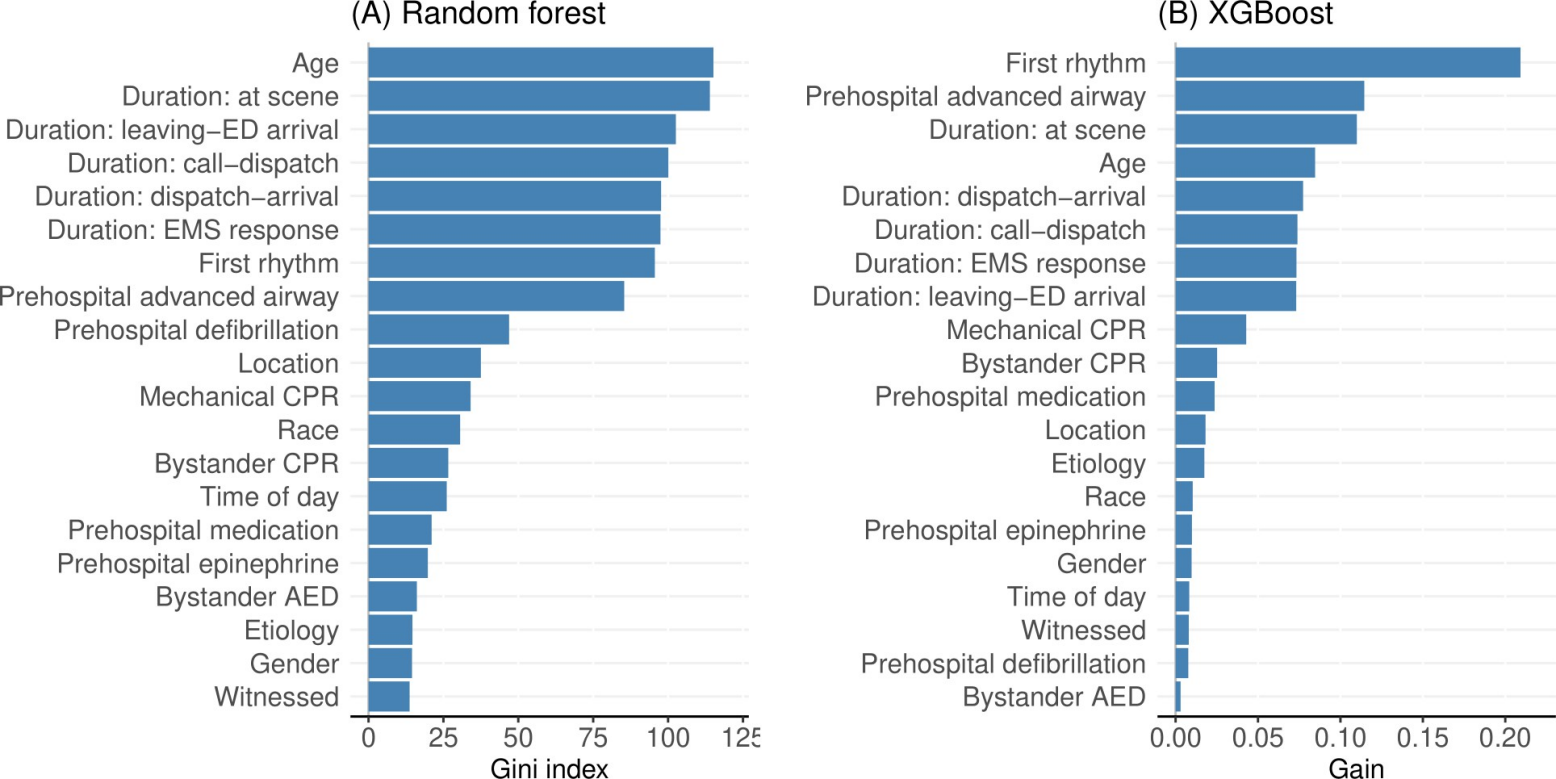
Variable importance based on (A) random forest and (B) XGBoost from the main analysis.

Unlike ShapleyVIC and logistic regression analyses, RF and XGBoost only reported relative importance values to rank all 20 variables (see Fig 3), without any measures of uncertainty to test statistical significance. Furthermore, the RF ranked all 6 continuous variables (i.e., age and the five variables for EMS system duration) at 1^st^ to 6^th^, higher than important categorical variables identified above, e.g., first rhythm, prehospital advanced airway, and witnessed arrest. The XGBoost performed slightly better than RF by ranking first rhythm and prehospital advanced airway as the 1^st^ and 2^nd^, but it still ranked the 6 continuous variables at 3^rd^ to 8^th^, higher than all other categorical variables. In view of these questionable findings, we did not include the RF and XGBoost in our simulated experiments.

### Prediction model development

A direct application of ShapleyVIC inference on variable importance is to filter out variables that may not strongly contribute (or may even add noise) to accurate prediction based on the 95% PI of overall variable importance. The traditional backward selection approach starting with all 20 variables developed a 11-variable logistic regression model (see Model A in Table 2). Similar to the logistic regression analysis of all 20 variables, Model A estimated significant association for race. The ShapleyVIC-assisted backward selection approach first excluded 7 variables with non-significant overall importance (including race), and subsequently used backward selection to develop a 9-variable model (see Model B in Table 2), where 8 variables were also included in Model A. When evaluated on the test data, Model A had an area under the receiver operating characteristic curve (AUC) of 0.860 (95% confidence interval [CI]: 0.840–0.879). Model B had a comparable AUC (0.861, 95% CI: 0.842–0.881) by using fewer variables and reasonably excluding race as a predictor. The two models had comparable classification performance when evaluated at the optimal threshold (see Table 3).

**Table 2:** Logistic regression models developed using traditional backward selection (Model A) and ShapleyVIC-assisted backward selection (Model B). For each model, the odds ratio (OR) and 95% confidence interval (CI) associated with each predictor was reported.

	Model A (11 variables) Adjusted OR (95% CI)	Model B (9 variables) Adjusted OR (95% CI)
(Intercept)	1.55 (0.81, 2.96)	1.69 (1.01, 2.85)
Age (10 years)	0.97 (0.97, 0.98)	0.98 (0.97, 0.98)
Location		
Home	0.55 (0.44, 0.69)	0.64 (0.51, 0.79)
Healthcare facility	1.08 (0.76, 1.52)	1.19 (0.84, 1.67)
Witnessed arrest	1.48 (1.16, 1.88)	1.38 (1.09, 1.75)
Bystander CPR	1.48 (1.20, 1.83)	1.53 (1.24, 1.89)
Shockable first rhythm	6.62 (5.36, 8.18)	4.96 (3.32, 7.42)
Mechanical CPR	0.54 (0.43, 0.67)	0.55 (0.44, 0.69)
Prehospital advanced airway	0.23 (0.18, 0.30)	0.23 (0.18, 0.30)
Prehospital medication	0.49 (0.40, 0.61)	0.52 (0.42, 0.63)
EMS response time	0.97 (0.94, 0.99)	--
Duration of ambulance at scene	1.04 (1.03, 1.06)	--
Race		
Indian	0.74 (0.53, 1.03)	--
Malay	0.64 (0.48, 0.86)	--
Others	1.07 (0.67, 1.70)	--
Prehospital defibrillation	--	1.38 (0.92, 2.08)

**Table 3:** Performance of Models A and B (see Table 2) evaluated on the testing data.

	Model A	Model B
AUC (95% CI)	0.860 (0.840, 0.879)	0.861 (0.842, 0.881)
Optimal threshold*	0.149	0.184
Accuracy (95% CI)	0.793 (0.738, 0.851)	0.833 (0.732, 0.870)
Specificity (95% CI)	0.795 (0.729, 0.846)	0.762 (0.704, 0.858)
Sensitivity (95% CI)	0.793 (0.755, 0.834)	0.820 (0.749, 0.849)
NPV (95% CI)	0.952 (0.941, 0.962)	0.948 (0.937, 0.965)
PPV (95% CI)	0.426 (0.383, 0.493)	0.467 (0.377, 0.525)

*Defined as the point nearest to the upper-left corner of the receiver operating characteristic curve.

CI: confidence interval; NPV: Negative Predictive Value; PPV: Positive Predictive Value.

## Discussion

Statistical modeling of associations between relevant factors and the outcome is an important way to understand underlying mechanisms of health-related outcomes and to uncover patterns for closer investigations in future studies. Compared to traditional regression analyses, ML methods are sometimes considered more powerful for such purposes due to their more complex and therefore more flexible model structures, and their promising predictive performance in some tasks. However, as highlighted in recent works [13,17–20], ML methods are not necessarily superior to traditional methods when working with structured and static clinical data. Complex ML models often require larger sample sizes to train and incur more cognitive burden when interpreting the findings. Hence, instead of directly applying ML models in clinical applications, it may be useful to enhance traditional analytical approaches using ML methods for more robust inference. The recently developed ShapleyVIC method for variable importance assessment is such an example, and has been demonstrated in applications for clinical big data [16]. This work highlights an additional benefit of this approach, i.e., robust performance with reduced sample sizes, which is valuable in clinical studies where large sample sizes are not always feasible. As illustrated in our example, the robustness of ShapleyVIC may also contribute to a more objective assessment of the role of sensitive variables (in our example gender and race) in clinical predictions for improved fairness.

An important consideration in clinical studies is to acquire sufficient data to assure sufficient statistical power to shortlist important variables to the outcome. As well understood by researchers and demonstrated in our simulated experiments, a reduction in sample size can attenuate statistical significance for some variables in regression analyses (not necessarily the lower-ranking ones), and with n = 500 we could only consistently find significance for two of 20 variables. By studying an ensemble of regression models, instead of a single logistic regression trained from the sample, ShapleyVIC findings were more stable when sample size reduced. In our simulated experiments, ShapleyVIC was generally consistent in shortlisting a set of important variables even when the sample size reduced to 500, although false positives became more likely for smaller sample sizes. In additional simulation studies, ShapleyVIC performed reasonably when the sample size further reduced to 250 but did not generate stable findings when n = 150 (see S1 Text for detail). Bootstrapping could be useful for assessing the stability of ShapleyVIC when working with such small samples.

In addition, the ensemble approach of ShapleyVIC avoids bias towards any single model when assessing variable importance, which has been demonstrated for reflecting the low and non-significant importance of race in a well-studied recidivism study [14]. This desirable property is demonstrated again in our analysis by estimating low importance for race and moderate importance for prehospital interventions, which is arguably more plausible than the opposite reported by the logistic regression. Such findings may provide an alternative perspective for understanding the role of sensitive variables in clinical risk prediction, where existing approaches (e.g., stepwise variable selection and RF; see Model A in Table 2 and Fig 3A) may report questionably high importance for race. Such complication may be avoided by excluding race from the analysis, but this approach misses the opportunity to obtain data-driven evidence for its role in the prediction.

The ensemble approach used in ShapleyVIC is commonly used in ML methods to boost performance and robustness, e.g., in the RF and XGBoost. However, as shown in our analyses, the complex mathematical structure of these methods can have unexpected impact on variable importance findings, e.g., reporting higher importance for continuous variables than categorical ones, which can confuse subsequent interpretation. ShapleyVIC avoids this by basing variable importance assessments on the familiar logistic regression to ensure general consistency with existing evidence generated using similar approaches. Specifically, in the main analysis (n = 4494) ShapleyVIC estimated significant overall importance for the following variables that were previously reported [21–24]: first rhythm, prehospital interventions (advanced airway, use of epinephrine or generally any medication, and defibrillation), age, arrest location, mechanical CPR, witnessed arrest, bystander CPR, EMS response time. The other two duration variables (ambulance dispatch to arrival, and emergency call to ambulance dispatch) had overall importance close to zero and were less reported in the literature. The wide 95% PI for gender indicates a high level of uncertainty regarding its importance, which may partially explain the contradictory findings on gender in different studies. When measuring variable importance, ShapleyVIC provides a unified measure for each variable that is directly comparable between continuous and categorical variables. Such comparison is less straightforward based on logistic regression analyses, as the regression coefficients can have small values for significant continuous variables and are reported on category level instead of variable level for categorical variables.

When there is sufficient data to develop prediction models, e.g., in our study with a cohort of 7490 subjects, ShapleyVIC contributes to an interpretable model development process in multiple ways. The commonly used stepwise model building methods, e.g., the backward selection used in our analysis, selects variables based on their impact on model performance when added to or excluded from a model. This may work reasonably well but provides limited insights on the importance of each variable to the outcome. More importantly, since such impact on model performance is evaluated by comparing single models with or without some variables, these traditional variable selection methods may be biased in a similar way as the conventional logistic regression analysis. For example, as discussed above, Model A developed using backward variable selection had the questionable finding of significant importance for race. By first investigating overall importance of all variables using ShapleyVIC and excluding variables with non-significant contribution, the ShapleyVIC-assisted backward selection reduced such bias, and reduced model size without affecting performance. It is also worth noticing that the resulting Model B had comparable predictive performance as a RF using all 20 variables (AUC = 0.861, 95% CI: 0.841–0.880), highlighting that complex black-box models do not necessarily bring practical benefits. In studies without sufficient samples to develop prediction models, the ShapleyVIC-assisted backward selection can help identify a more focused list of variables for further investigation.

In this study, we demonstrated several preferable properties of ShapleyVIC compared to existing variable importance methods. Due to the intensive computation involved in calculating Shapley values and the need to calculate such values for multiple (in our example 250) regression models, a practical limitation of ShapleyVIC is the long computation time (approximately 17 hours for our main analysis, depending on device specifications; see Methods for detail) that can increase considerably with the number of variables. In our analysis of variable importance for OHCA survival, a limitation is that we did not include some potential predictors (e.g., medical history, and timing, duration and quality of CPR) due to insufficient information. Prediction models reported in this work are only for demonstration purpose and not for clinical applications.

While many existing works discuss the replacement of traditional analytical approaches by ML methods, in this work we have demonstrated the benefits of an integrative ShapleyVIC method for variable importance assessment, which combines regression-based analysis with ML techniques for robust performance with reduced bias, and works reasonably well even with just a few hundred observations. Hence, we propose ShapleyVIC as a preferable alternative to current practice in variable importance assessment, which is not only useful for analyzing clinical big data (as previously demonstrated [16]), but also helps hypothesis testing or exploratory analyses with limited data (as shown in our simulated experiments). Although ShapleyVIC does not directly assess or improve fairness, its robust and less biased estimation of variable importance may contribute to the development of fairer clinical risk prediction models. Future work will further evaluate this potential contribution of ShapleyVIC in more diverse application contexts.

## Materials and methods

### Ethics statement

This study used nationwide data in Singapore collected between 2009 and 2019 through the Pan-Asian Resuscitation Outcome Study (PAROS), approved by the SingHealth Centralised Institutional Review Board (2013/604/C) and Domain Specific Review Board (2013/00929). The waiver of informed consent was approved for the collection of data.

### Study design and setting

We included OHCA patients aged 18 years or above, who were transported by EMS and had resuscitation attempted, had non-trauma etiology, and achieved ROSC. The binary outcome of interest was survival to discharge, or 30 days if not yet discharge. Twenty variables were included covering information on patient demographics, OHCA incidence, EMS system duration, and prehospital interventions:

- Patient demographics: age, gender, and race.
- Information on OHCA incidence: witnessed arrest, cardiac etiology, shockable initial rhythm, time of day, and arrest location.
- EMS system duration: time between emergency call and EMS arrival [EMS response time], and various ambulance response times (time between ambulance dispatch, arrival at scene, leaving scene, and arrival at the emergency department).
- Prehospital interventions received: bystander CPR, bystander AED, mechanical CPR, defibrillation, advanced airway, use of epinephrine, and use of any medication.

### Variable importance assessment

We assessed variable importance using the ShapleyVIC method [14], which improves upon straightforward regression-based inference by studying an ensemble of regression models. Specifically, in this study we first trained a conventional multivariable logistic regression of all 20 variables on the training set (i.e., the optimal model for the current cohort), and randomly generated 250 logistic regression models centered around the optimal model that had near-optimal performance (where the model loss exceeded the minimum level by <5%). Variable importance to each of the 250 model was assessed using Shapley values [25], and was summarized across models via meta-analysis to estimate the overall importance and 95% prediction interval (PI). Significant overall importance was indicated by 95% PI entirely above zero. As will be empirically demonstrated in this work, by actively including nearly optimal models when estimating overall variable importance, ShapleyVIC partially accounts for the shifts in regression coefficients due to sampling variability, hence generating more stable findings.

We compared ShapleyVIC with conventional multivariable logistic regression analysis, where we ranked relative variable importance by p-values and used absolute values of regression coefficients as complementary measures of variable impact on predictions. Additionally, we included two commonly used ML methods, the RF and XGBoost, and reported Gini index from the RF [7] and fractional contribution of variables from XGBoost (in terms of total gain of each variables’ splits) [8] as measures of relative variable importance.

### Prediction model development

The backward selection method used in this study refers to stepwise variable selection using the Akaike information criterion (AIC) starting with all candidate variables. This method is widely used for developing prediction models in clinical research [26], which results in parsimonious regression models that are straightforward to interpret. We complement the backward selection approach with a variable pre-screening step based on the ShapleyVIC inference to eliminate variables with low contribution to accurate prediction, referred to as ShapleyVIC-assisted backward selection, and compared it with the conventional backward selection approach.

### Statistical analysis

Patient characteristics were described using frequency and percentage for categorical variables, and median and 1^st^ and 3^rd^ quartiles for continuous variables. The ShapleyVIC method was jointly implemented by a Python library (to generate an ensemble of regression models and compute variable importance) and an R package (to pool information across the ensemble via meta-analysis and visualize the findings) [27]. Technical details on ShapleyVIC configuration is described in the next subsection. All other methods were implemented in R, using the *randomForest* package for the RF [28], *xgboost* package for the XGBoost [29], and *MASS* package for backward selection [30]. Analyses were conducted using R (version 4.2.0) [31] and Python 3.10.9.

### ShapleyVIC configuration

#### Sample size

ShapleyVIC consists of three major steps: (i) train an optimal model, (ii) generate a group of nearly optimal model, and (iii) compute Shapley values from each nearly optimal model for overall importance assessment. Using unnecessarily large sample size in step (iii) increases computational time. In our main analysis with a training set of n = 4494, we used the full training set in steps (i) and (ii), and randomly selected half of the training set (n = 2247) as the explanation data in step (iii), which we found sufficient to obtain stable results in preliminary experiments. In simulated experiments with n = 2500 and n = 500, we used the full sample in all three steps due to limited sample sizes.

#### Generating nearly optimal models

As detailed in the previous methodological work [14], nearly optimal logistic regression models were generated based on the multivariable normal distribution of regression coefficients from the optimal model, but the variance-covariance matrix is randomly scaled (with scaling parameter bounded between *u*_*1*_ and *u*_*2*_) to sufficiently explore the model space. Suitable values for *u*_*1*_ and *u*_*2*_ need to be tuned by inspecting the proportion of sampled models with eligible performance (i.e., where the model loss exceeded the minimum level by <5%), preferably close to 70%–80%. In our main analysis and simulated experiments, we used *u*_*1*_ and *u*_*2*_ values automatically selected by the *init_hyper_params* function of the ShapleyVIC Python library. Technical details on software implementation of ShapleyVIC is described in our online guidebook (https://nliulab.github.io/ShapleyVIC/). In previous studies [14,16] we used 350 nearly optimal models for detailed variable importance assessment, but in this study we found in preliminary experiments that 250 models are generally sufficient for estimating overall variable importance.

#### Computational time

In addition to the number of variables and size of explanation data, computational time is also affected by computer specification, e.g., hardware and operating system. In our main analysis that included 20 variables (24 columns after one-hot encoding of categorical variables), the ShapleyVIC algorithm took approximately 17 hours to complete, by using 15 cores in parallel computing on a PC with 40 cores in total (Windows 10 Education; Intel(R) Xeon(R) Silver 4210 CPU @ 2.20GHz 2.19GHz (2 processors); 128GB RAM).

## Supporting information

S1 Text Supplementary Material. (PDF)
