# Supplementary material for: Variable importance analysis with interpretable machine learning for fair risk prediction
Source: PLOS Digit Health. 2024 Jul 12;3(7):e0000542. doi: 10.1371/journal.pdig.0000542 (PMC11244764; doi:10.1371/journal.pdig.0000542)

Supplementary Material

We performed additional simulation studies with sample sizes n=250 and n=150. Two pairs of variables were strongly correlated, i.e., prehospital epinephrine & prehospital medication, and shockable first rhythm & prehospital defibrillation, and such strong correlation affected the convergence of logistic regression models when n=250 and n=150. Therefore, in these additional simulation studies we excluded prehospital medication and prehospital defibrillation. When n=150 we further excluded bystander automated external defibrillator (AED) which affected the convergence of the logistic regression models, and dichotomized race (Chinese vs non-Chinese), location (public vs non-public) and time of arrest (6AM-7PM vs other time periods) to improve model stability.

For each sample sizes, we generated 5 random samples from the full cohort of n=7490 subjects. We analyzed variables importance (18 variables when n=250 and 17 variables when n=150) using ShapleyVIC as described in Methods of the main text, and used all samples to generate nearly optimal models and evaluate variable importance. These additional simulation studies were performed on a MacBook Air with Apple M2 chip (8 cores, 16 GB memory), by using 7 cores in parallel. The ShapleyVIC algorithm on average took 10 minutes when n=250 and 5 minutes when n=150.

Results from the ShapleyVIC analysis are summarized in Fig S1 below, with variables arranged in the same order as in the main analysis for convenient comparison. When n=250, findings from ShapleyVIC were generally similar to those when n=500, but with some elevated chances for false positives (e.g., Fig S1A). When n=150, stability of the ShapleyVIC findings further decreased (e.g., Fig S1F-S1G) and may not be reliable for inference. In view of the reasonably short computational time with such small sample sizes, when applying ShapleyVIC to small samples, researchers may consider performing multiple analyses on bootstrap samples from the original data to assess stability of results.

Fig S1. Variable importance analysis from ShapleyVIC and logistic regression analyses in additional simulation experiments with n=250 and n=150, arranged by the same variable ordering as in the main analysis (see Fig 1 in the main text). Blue color and "*" indicate significant variable importance, i.e., 95% prediction interval above zero for ShapleyVIC.

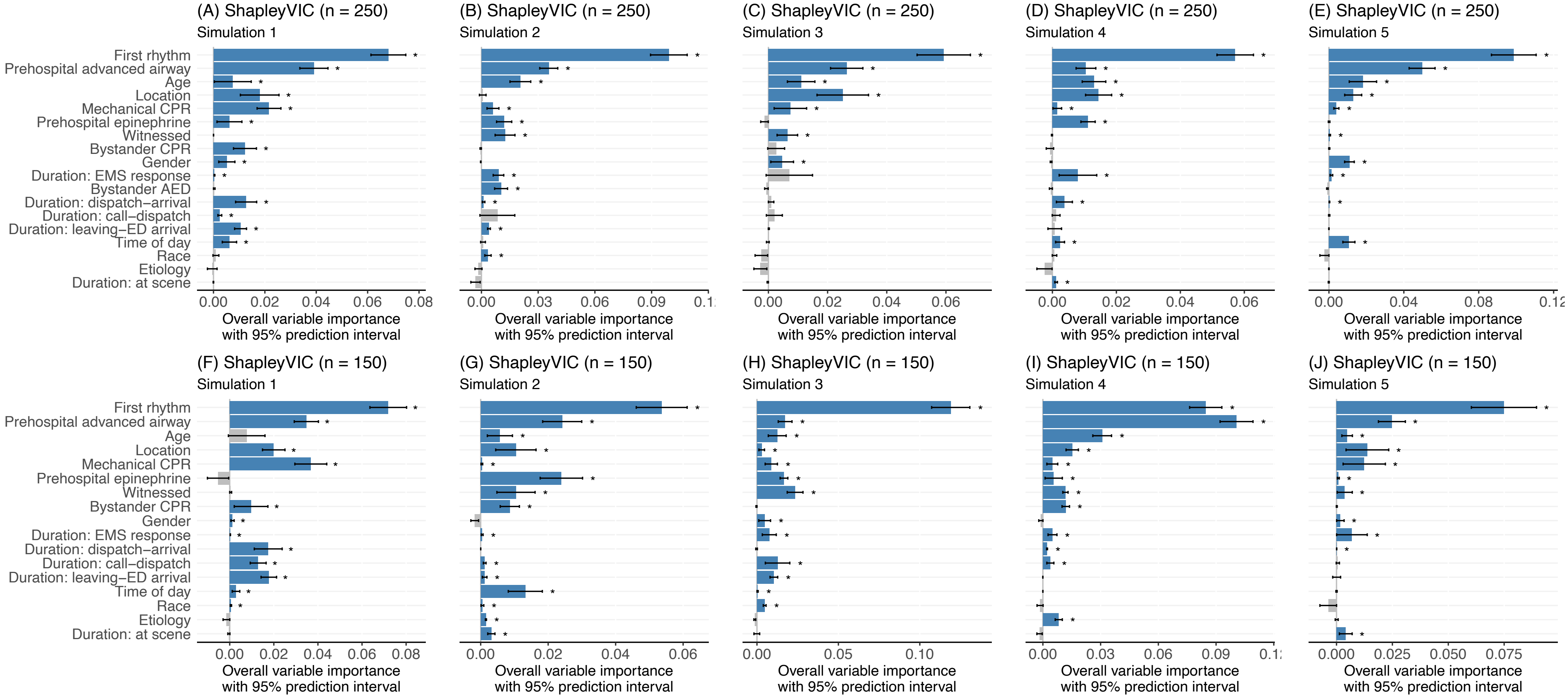

Supplement: S1 Text — (PDF) [file pdig.0000542.s001.pdf]
